# Advances in the Understanding of the Pathogenesis of Triple‐Negative Breast Cancer

**DOI:** 10.1002/cam4.70410

**Published:** 2024-11-19

**Authors:** Yuhan Liu, Yuhan Zou, Yangli Ye, Yong Chen

**Affiliations:** ^1^ School of Clinical Medicine Shandong Second Medical University Weifang China; ^2^ College of Life Sciences and Technology Shandong Second Medical University Weifang China; ^3^ Key Laboratory of Immune Microenvironment and Inflammatory Disease Research in Universities of Shandong Province, School of Basic Medical Sciences Shandong Second Medical University Weifang China

**Keywords:** classification, drug resistance, genomic alterations, signaling pathways, triple‐negative breast cancer

## Abstract

**Background:**

Triple‐negative breast cancer (TNBC) is a heterogeneous disease characterized by high aggressiveness, high malignancy, and poor prognosis compared to other breast cancer subtypes.

**Objective:**

This review aims to explore recent advances in understanding TNBC and to provide new insights and potential references for clinical treatment.

**Methods:**

We examined current literature on TNBC to analyze molecular subtypes, genetic mutations, signaling pathways, mechanisms of drug resistance, and emerging therapies.

**Results:**

Findings highlight key aspects of TNBC's molecular subtypes, relevant mutations, and pathways, alongside emerging treatments that target drug resistance mechanisms.

**Conclusion:**

These insights into TNBC pathogenesis may help guide future therapeutic strategies and improve clinical outcomes for patients with TNBC.

## 1 Introduction

Breast cancer is the most common malignancy among women. Tumors characterized by the absence of estrogen receptors (ER), progesterone receptors (PR), and human epidermal growth factor receptor 2 (HER2) are classified as triple‐negative breast cancer (TNBC), accounting for approximately 15%–20% of all breast cancer cases [1]. Compared to hormone receptor‐positive or HER2‐positive breast cancers, TNBC is distinguished by an aggressive clinical course, with earlier onset, high metastatic potential, and poor clinical prognosis, often manifesting as high recurrence rates and low survival rates.

This review summarizes recent advances in the classification of TNBC, key genetic mutations, affected signaling pathways, and mechanisms of drug resistance.

## 2 Subtype Classification of Triple‐Negative Breast Cancer Based on Gene Expression Profiles

To gain a deeper understanding of TNBC heterogeneity, Lehmann et al. [2] in 2011 proposed a six‐subtypes classification based on gene expression profiles: basal‐like 1 (BL1), basal‐like 2 (BL2), immunomodulatory (IM), mesenchymal (MES), mesenchymal stem‐like (MSL), and luminal androgen receptor (LAR) subtypes. In subsequent studies, Lehmann et al. [3] refined this classification into four primary subtypes: BL1, BL2, M, and LAR. Building upon the Lehmann classification, Burstein et al. [4] conducted a more detailed analysis in 2015 using RNA and DNA profiling, categorizing TNBC into four subtypes: LAR, mesenchymal (MES), basal‐like immunosuppressed (BLIS), and basal‐like immune‐activated (BLIA). In 2019, Fudan University researchers [5] further refined the classification into four subtypes: LAR (23%), IM (24%), BLIS (39%), and MES (15%).

The LAR, BLIS/BLA, and MES subtypes have been extensively investigated in TNBC heterogeneity research. The LAR subtype exhibits lower genomic complexity, with mutations predominantly affecting PIK3CA, AKT1, NF1, GATA3, and CDH1. The BLIS subtype demonstrates a high frequency of TP53 mutations and HRD. The MES subtype is characterized by epithelial‐to‐mesenchymal transition (EMT), high expression of tumor stem cell‐related genes, and elevated aberrations in the PI3K/AKT/mTOR pathway. Lehmann's original six‐subtype classification provided a comprehensive understanding of distinct molecular pathways. The 2016 update further enhanced the classification's clinical utility. Burstein classification emphasized the immune microenvironment, linking each subtype to specific therapeutic targets. This approach, particularly the stratification of immune status (BLIS and BLIA) and highlighting androgen receptor signaling (LAR), provides clear guidance for personalized treatment. The Fudan classification is distinguished by its detailed stratification of the immune microenvironment and tumor molecular characteristics, particularly in Asian populations, retaining the immune regulatory subtype. This classification not only enables more precise TNBC subtyping but also provides new insights and rationale for the clinical selection of immunotherapy and targeted therapies.

## 3 Somatic Genomic Alterations in TNBC

### 3.1 Gene Mutations

Through whole‐genome sequencing, whole‐exome sequencing, and targeted sequencing technologies, an in‐depth genomic analysis of the TNBC genome has revealed its remarkable complexity, including a high mutation burden and extensive gene copy number variations [6, 7, 8]. The most frequently mutated gene is TP53 (over 80%), followed by PIK3CA. Other low‐frequency mutated genes (< 5%) include PTEN, KMT2C, and RB1 [7, 9]. The copy number alterations (CNAs) in TNBC are complex, including gains in 1q, 8q, and 10q, losses in 5q and 8p, as well as amplifications of EGFR and FGFR2, and loss of PTEN [10, 11].

Curtis et al. [11] proposed 10 integrative clusters (IntClust 1–10) by combining CNAs and gene expression profiles. Among these, IntClust 10 primarily consists of poorly differentiated TNBC with high TP53 mutations and moderate genomic instability, characterized by the loss of chromosome 5q. The IntClust 4 subtype exhibits low genomic instability and lacks CNAs, with twice the number of deletions at the TRG and TRA TCR loci compared to other subtypes (except IntClust 10). These deletions at TCR loci are associated with lymphocyte infiltration [11], suggesting an interaction between genetic alterations in tumor cells and their microenvironment.

Notably, different TNBC subtypes exhibit distinct CNA profile characteristics [12]: the BL1 subtype involves gains such as MYC, PIK3CA, and CDK6, alongside losses in BRCA2, PTEN, MDM2, RB1, and TP53. The LAR subtype shows gains in EGFR and AKT1 and losses in genes like CCND3, AKT2, and ESR1. The M subtype is significantly associated with gains in DNMT3A and TP53 and losses in PDGFRA, RB1, and MAP3K1 [12]. Moreover, the BLIS subtype frequently exhibits amplifications at 9p23 (61%) and 12p13 (74%) [5].

Furthermore, research has identified key pathways involved in TNBC‐specific somatic mutations and epigenomic alterations, including retinal biosynthesis, BAG2, LXR/RXR, EIF2, and P2Y purinergic receptor signaling pathways [13].

### 3.2 Mutational Signatures

Multiple studies [14, 15, 16, 17, 18] have demonstrated that somatic genetic alterations in breast cancer, particularly passenger mutations, provide insights into cancer genome evolution, dysfunction, and potential therapeutic targets. Mutational signatures analysis has identified aging or clock‐like mutations, APOBEC‐mediated mutations, and HR DNA repair deficiencies as the most common mutational processes in breast cancer [5, 8, 14, 15]. Notably, the HRD signatures is more enriched in TNBC [5, 8, 19].

Two types of indel mutations and six rearrangement signatures (RS‐1 to RS‐6) have been identified in breast cancer research [8]. Among these, RS‐1 and RS‐3 are closely associated with tandem duplications (TDs). Specifically, in TNBC patients lacking BRCA1 mutations or promoter hypermethylation, tumors often exhibit large‐scale (over 100 kb) tandem duplication regions associated with RS‐1. Conversely, in more than 90% of TNBC cases with BRCA1 mutations or high methylation features, smaller‐scale (less than 10 kb) tandem duplications linked to RS‐3 and HRD are observed [8, 18, 19]. Additionally, in high‐grade TNBC, approximately 52.8% exhibit a tandem duplication phenotype (TDP), likely resulting from the interaction between genomic instability and replication errors in TNBC [20]. Based on the size of TDs, TDPs can be categorized [8]. Specifically, small TDs (~10 kb) are enriched in BRCA1‐mutant TNBC [20], affecting tumor suppressor genes such as PTEN and RB1 [21]. Medium‐sized TDs (~50–600 kb) and large TDs (~2 Mb) are associated with the activation of pathways involving CCNE1 and CDK12, respectively [20, 21].

Another genomic feature identified in TNBC is whole‐genome duplication (WGD). WGD originates from tetraploidization, which buffers the detrimental effects of a high mutation burden and mitigates Muller's ratchet effect, thereby influencing certain biological characteristics of TNBC [22, 23]. Approximately 45% of patients exhibit at least one instance of WGD [24, 25], which is associated with the incidence of somatic CNAs [25] and tumor mutation burden [22, 23]. WGD occurs frequently in certain TNBC subtypes, such as BLIS [5], and may play a critical role in tumor evolution [26].

### 3.3 Homologous Recombination DNA Repair Deficiencies in TNBC

In TNBC, deficiencies in double‐strand DNA repair are primarily driven by mutations in BRCA1 and other homologous recombination (HR)‐related genes [27]. Although only 19% of TNBC cases harbor BRCA1 germline mutations, the majority of TNBCs exhibit a BRCA‐like HRD phenotype [8] (BRCAness [28]), which is associated with variations in other HR genes such as PALB2, CHEK2, ATM, and NBN [29]. Overall, approximately 14.6% of TNBCs carry mutations in HRD‐related genes, with BRCA1 (8.5%), BRCA2 (2.7%), and other HRD genes (3.7%) [30]. Research by Polak et al. [31] found that in TNBCs with high HRD activity, 35% of cases show BRCA1/2 biallelic loss, whereas the remaining cases exhibited BRCAness, with mutations in RAD51C, PALB2, and BARD1 representing 13% of cases [32, 33]. Based on the HRDetect method, TNBC can be classified into three subgroups: HRDetect‐high, HRDetect‐intermediate, and HRDetect‐low. The HRDetect‐high group demonstrates better prognosis and high sensitivity to chemotherapy; the HRDetect‐intermediate group is significantly enriched with CCNE1 amplification; and the HRDetect‐low group primarily exhibits abnormalities in the PIK3CA/AKT1 pathway [34]. These findings provide important insights for personalized treatment strategies in TNBC.

## 4 Key Signaling Pathways Associated With the Development and Progression of TNBC

### 4.1 PI3K/AKT/mTOR Pathway

The PI3K/AKT/mTOR (PAM) pathway is responsible for regulating cell cycle progression, metabolism, angiogenesis, and responses to stimuli such as growth factors, metabolites, and hypoxia. Aberrations in the PI3K/AKT/mTOR pathway are among the central carcinogenic mechanisms in TNBC [7, 35].

In TNBC, overexpression of upstream regulators such as EGFR, activating mutations in PIK3CA, and reduced expression of PTEN and proline‐rich inositol polyphosphatase are closely associated with the oncogenic activation of the PAM pathway [36, 37]. However, activating mutations in downstream molecules of PI3K (i.e., AKT and mTOR) and in homologous pathways (MAPK and RAS) are rare in TNBC [7].

Research has shown that long non‐coding RNAs (lncRNAs) are critical determinants of malignancy downstream of the PI3K pathway, with LINC01133 identified as an important effector of PI3K‐AKT signaling. LINC01133 activates AKT in a PI3K‐independent manner by regulating the mTORC2 pathway and competitively upregulating PROTOR1/PRR5 through hnRNPA2B1, which is closely associated with poor survival rates in TNBC [38].

### 4.2 Notch Pathway

The Notch signaling pathway is a highly conserved cellular signaling mechanism that plays crucial roles in cell proliferation, differentiation, stem cell maintenance, and EMT—processes that are integral to tumorigenesis. This pathway comprises four Notch receptors (Notch1‐4) and five ligands (Jagged‐1, Jagged‐2, Delta‐1, Delta‐3, and Delta‐4). Notch signaling activation occurs through the binding of Notch receptors to their ligands, mediated by TACE/ADAM proteases, and the γ‐secretase complex [39].

In breast tissue, Notch signaling is essential for development and homeostasis. Its dysregulation can promote breast cancer onset [40], with aberrant activation often representing an early event in breast cancer progression [41]. Numerous studies have linked Notch1 signaling with the development and metastasis of TNBC [42, 43]. Notch1 exacerbates TNBC development through a mitochondrial fission‐positive feedback loop [44], while also activating the ATR‐CHK1 signaling pathway to restore the S/G2 and G2/M cell cycle checkpoints, thereby suppressing mitotic catastrophe induced by BRCA1 deficiency and promoting TNBC development [45]. Additionally, Notch1 accelerates TNBC progression by facilitating epithelial‐mesenchymal transition (EMT) and regulating the cell cycle [45].

High expression of Notch2 is associated with improved survival rates in breast cancer patients [46]. However, its specific role in breast cancer remains unclear [47, 48, 49]. Notch3 is overexpressed in TNBC [50] and is closely linked to malignant phenotypes and accelerated cell proliferation [51], while also enhancing the invasive and metastatic capabilities of TNBC [52]. Moreover, the expression level of Notch3 is significantly associated with EGFR, influencing both chemoresistance and resistance to targeted therapies [53, 54]. Additionally, Notch4 overexpression is also associated with TNBC [55], particularly in metastatic cases [56]. Notch4 levels are critical for breast cancer stem cell self‐renewal and chemoresistance [57].

Recent studies have demonstrated the multifunctional roles of Notch receptors in TNBC [58], With Notch1 and Notch2 acting as tumor promoters, whereas the subcellular localization of Notch3 and Notch4 is associated with prognostic factors. Therefore, assessing the subcellular localization of Notch receptors is crucial [58].

These data underscore that the specific roles of Notch receptors in TNBC warrant further investigation.

### 4.3 JAK2/STAT3 Pathway

The JAK2/STAT3 pathway is composed of the nonreceptor tyrosine kinase Janus kinase 2 (JAK2) and the signal transducer and activator of transcription (STAT) proteins [59]. JAK2 is involved in various signaling processes, whereas STAT3 regulates cell proliferation, differentiation, and apoptosis upon nuclear translocation, exerting significant influence in oncogenic inflammatory microenvironments [60]. Abnormal activation of the JAK2/STAT3 signaling pathway has been implicated in the onset, angiogenesis, metastasis, and development of resistance in TNBC [59].

Iron overload induces the expression of IL‐6, which subsequently activates the JAK2/STAT3 signaling pathway, promoting the malignant transformation of TNBC cells [61]. Additionally, HOXC10 contributes to breast cancer progression by activating the IL‐6/JAK2/STAT3 signaling axis [62].

Notably, activation of TrkA enhances STAT3 activity, promoting the transcription of related genes in TNBC and HER2‐overexpressing breast cancers, thereby supporting the growth of breast cancer stem cells [63]. Conversely, vascular endothelial growth factor‐alpha (VEGF) upregulates the expression of Myc and Sox2 through its receptor VEGFR‐2, thereby enhancing the self‐renewal capacity of breast cancer stem cells via the JAK2/STAT3 pathway [64].

Research has also identified inhibitory strategies targeting the JAK2/STAT3 pathway. For instance, the redox enzyme Wwox [65] and Glyceryl trinitrate (GTN) [59] can inhibit this pathway, thereby curtailing TNBC metastasis and proliferation. Moreover, the natural alkaloid piperlongumine (PL), either alone or in combination with doxorubicin, demonstrates anti‐cancer effects by inhibiting the JAK2/STAT3 pathway [66]. These findings suggest that JAK2/STAT3 represents a potential therapeutic target in the treatment of TNBC.

### 4.4 Wnt/β‐Catenin Pathway

The Wnt/β‐catenin signaling pathway, also known as the canonical Wnt pathway, is involved in physiological processes including embryonic development, stem cell homeostasis, tumorigenesis, and drug resistance [67]. Increased Wnt pathway activity is often associated with enhanced stem cell potential and more aggressive forms of cancer [68], playing a critical role in maintaining breast cancer stem cell characteristics [69].

Activation of the Wnt/β‐catenin signaling pathway is linked to the accumulation of nuclear β‐catenin, which promotes cell migration, colony formation, and chemoresistance in TNBC cells [70, 71]. Tumors exhibiting a nuclear β‐catenin phenotype or loss of membrane β‐catenin are more likely to be diagnosed as TNBC [72], which typically lacks mutations in Wnt/β‐catenin pathway‐related proteins. Instead, pathway dysregulation is mediated by β‐catenin‐driven transcriptional activation [71, 73] and overexpression of associated mediators such as Frizzled [74] and LRP6 [75]. LRP6 is associated with cell migration and invasion [76], whereas elevated FZD7 promotes tumorigenesis through the transcription factor p63 [74, 77].

Recent studies have revealed new mechanisms of β‐catenin activation in TNBC: upregulation of SHC4 enhances Src kinase autophosphorylation, subsequently regulating the β‐catenin pathway and promoting TNBC metastasis [78]. METTL3 regulates FAM83D protein expression through m6A modification, exacerbating TNBC progression [79]. Additionally, KRT17 and Wnt/β‐catenin signaling have been identified as negative regulators of APC and AXIN1, and interference with KRT17 can reverse TNBC cell resistance to doxorubicin through modulation of the Wnt/β‐catenin pathway [80].

### 4.5 Hedgehog Pathway

The Hedgehog (Hh) signaling pathway is composed of three ligands (Sonic hedgehog [Shh], Desert hedgehog [Dhh], and Indian hedgehog [Ihh]), the transmembrane receptor Patched (PTCH), and the G protein‐coupled receptor Smoothened (SMO) [81].

The Hh signaling pathway is involved in cancer cell invasion, metastasis, drug resistance, and tumor recurrence following treatment [82]. In TNBC, overexpression of the Shh ligand is associated with poorer survival outcomes [83]. The basal expression levels of GLI1/2 are higher in TNBC compared to HR‐positive breast cancer [84]. Furthermore, GLI1 knockout reduces the viability of breast cancer cells, and high expression of GLI1 in HR‐negative breast cancer is indicative of poor prognosis [85].

**FIGURE 1:**
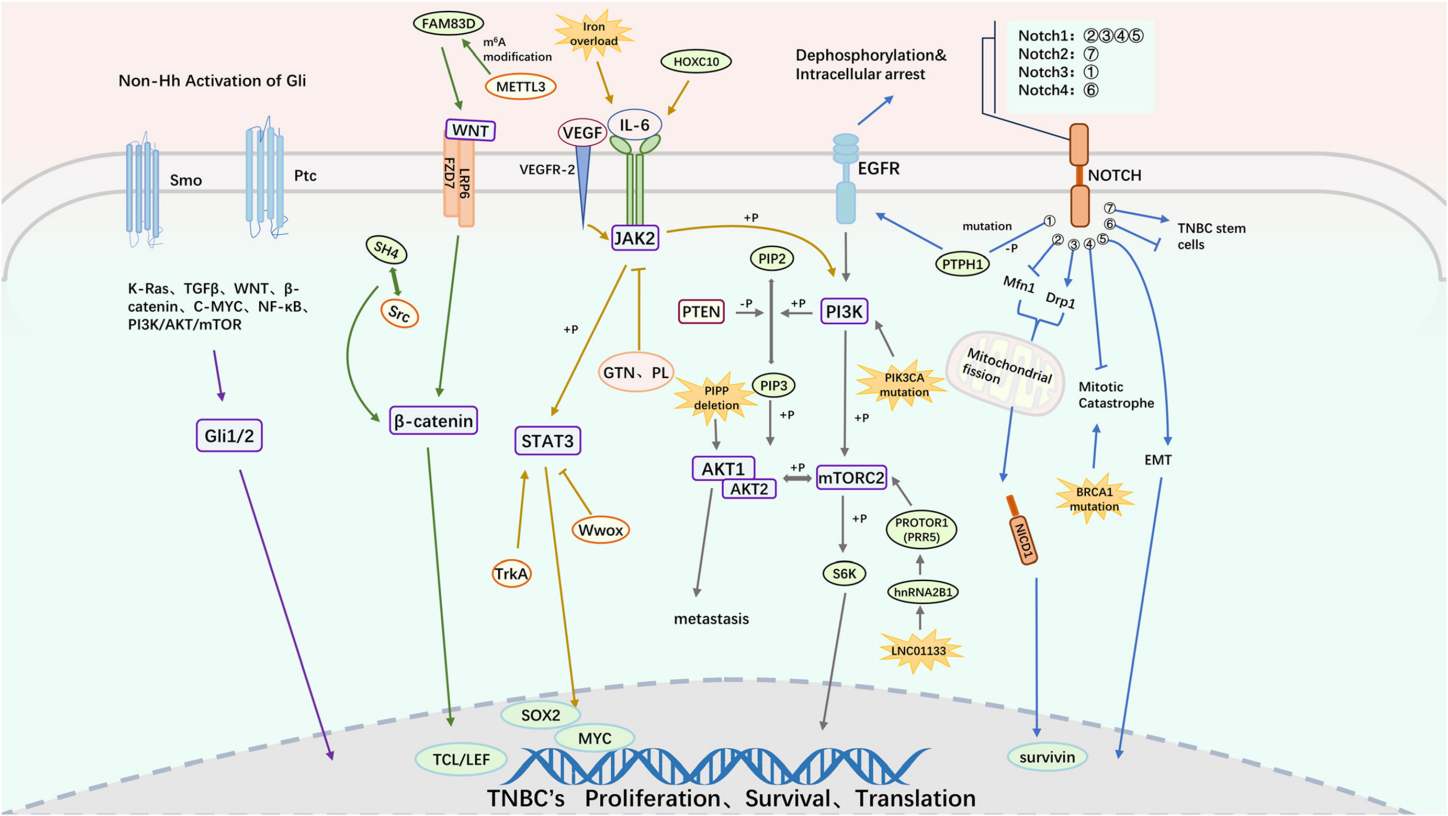
The signaling pathways involved in drug resistance in TNBC.

When the expression of Hh ligands is low, elevated levels of GLI1/2 suggest atypical activation [86]. Atypical Hh activation can be observed downstream of oncogenic pathways, such as PI3K‐Akt‐mTOR [87], K‐Ras, c‐Myc, Wnt‐β‐catenin, and TGFβ [88], mediated through transcriptional upregulation of GLI1… Abnormal transcriptional upregulation of GLI1 can also be observed downstream of NF‐κB in claudin‐low breast cancer [89]. Therefore, the increased expression of GLI1/2 in atypical pathways represents a primary mechanism of Hh pathway activation in TNBC. Additionally, the Hh pathway in TNBC promotes stem cell populations [84], activates cancer‐associated fibroblasts [90], enhances cancer cell invasiveness [91], and facilitates angiogenesis [92] through the upregulation of GLI1/2 transcription (Figure 1).

## 5 Mechanisms of Chemotherapy Resistance in TNBC

### 5.1 Chemotherapy Resistance to Platinum‐Based Agents

Cisplatin, a key drug in TNBC treatment, functions by inserting and cross‐linking DNA, thereby disrupting RNA transcription, and DNA replication, leading to cell cycle arrest and apoptosis [93, 94]. However, cisplatin resistance significantly impairs its clinical efficacy. Non‐coding RNAs, genetic mutations, and specific proteins play critical roles in the mechanisms underlying cisplatin resistance.

Research indicates that various miRNAs (e.g., miR‐105 and miR‐93‐3p) [95, 96, 97, 98] and lncRNAs (e.g., DLX6‐AS1 and DANCR) [99, 100, 101] influence cisplatin sensitivity by regulating pathways like Wnt/β‐catenin and GDF11. Additionally, circRNAs, such as circUBAP2 [102] and hsa_circ_0000199, [103] contribute to cisplatin resistance by modulating signaling pathways.

**FIGURE 2:**
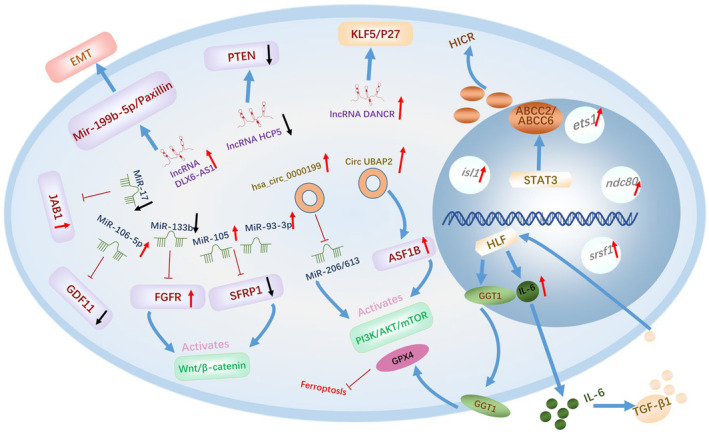
Mechanisms of chemoresistance to platinum‐based therapy in TNBC.

Mutations in BRCA1/2 genes and HRD are associated with increased cisplatin sensitivity [104]. Furthermore, mutations in genes such as NDC80 and ISL1 are closely related to cisplatin resistance [105, 106, 107, 108]. Specific proteins, including Lifeguard, Notch1 and GGT1, promote resistance by inhibiting apoptosis or modulating oxidative‐reductive mechanisms [109, 110, 111]. Moreover, dexamethasone induces cisplatin resistance by upregulating KLF5 [112], and transcription factors like STAT3 [113] and HLF [114] are also involved in cisplatin resistance (Figure 2).

### 5.2 Chemotherapy Resistance to Taxanes

Taxanes, such as paclitaxel (PTX), are crucial for TNBC treatment due to their ability to stabilize microtubules and inhibit mitosis [115]. However, the development of taxane resistance limits their long‐term efficacy.

The BRCAness phenotype significantly affects the response of TNBC patients to taxane‐based chemotherapy (e.g., paclitaxel, docetaxel). BRCA1‐like scores are weakly positively correlated with docetaxel sensitivity [104], and patients with BRCAness exhibit a lower response rate to neoadjuvant taxane chemotherapy, suggesting that BRCAness could serve as a potential biomarker for taxane resistance [116].

Studies have revealed that while paclitaxel induces cell death, it also activates resistance pathways involving genes such as IL‐6, CXCL8, and TP53 mutations [117]. Novel resistance genes like SYTL4 [118], and the identification of paclitaxel‐resistant subpopulations (e.g., AKR1C3+, IDO1+, HEY1+) and key transcription factors (e.g., STAT1, CEBPB) through scRNA‐seq, further elucidates the complexity of the resistance mechanisms [119].

Noncoding RNAs also play a significant role in taxane resistance. Specific miRNAs (such as miR‐186‐5p and miR‐5195‐3p) [120, 121, 122] and circRNAs (such as Circ‐ABCB10 and Circ‐RNF111) [123, 124, 125, 126, 127] are involved in resistance by regulating relevant pathways.

Furthermore, proteins such as the HDAC9 truncation isoform MITR [128], SERPINE1 [129], and TNFSF13 [130] promote resistance, whereas actin‐regulating protein MENA [131], IL22 [132], LIN9 [133], and glycolysis‐related molecules such as TRAF6 and PKM2 also participate in the regulation of paclitaxel resistance [134].

**FIGURE 3:**
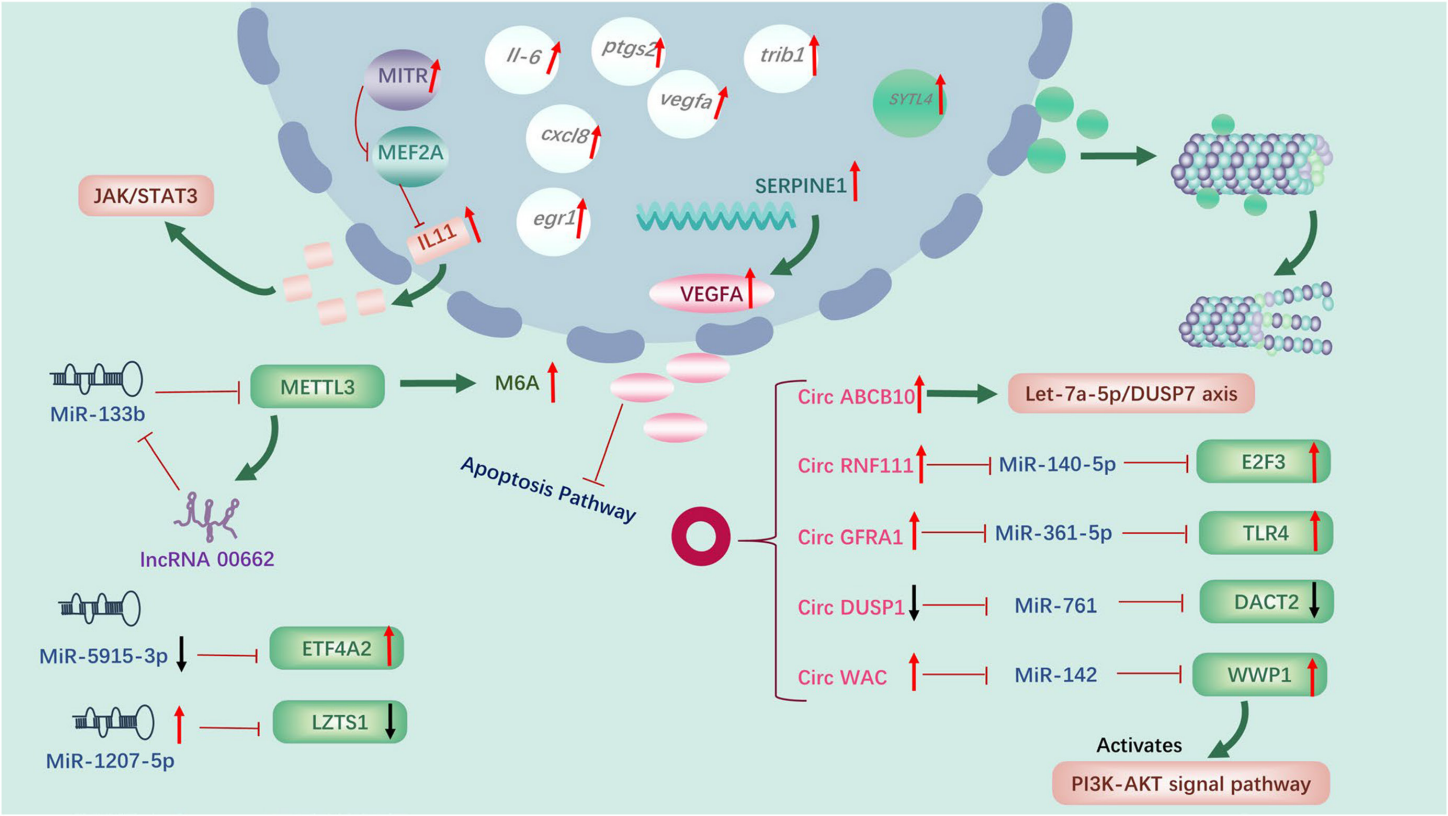
Mechanisms of chemoresistance to taxane‐based therapy in TNBC.

In summary, paclitaxel resistance in TNBC involves a variety of genes, noncoding RNAs, and signaling pathways, highlighting the need for further research and the exploration of new therapeutic strategies (Figure 3).

### 5.3 Chemotherapy Resistance to Anthracyclines

Anthracycline, such as doxorubicin (ADM), affect DNA topoisomerase activity, impede DNA replication, and are a frontline treatment for TNBC [135]. However, long‐term use often leads to drug resistance.

Long‐term exposure of TNBC cells to ADM induces significant changes in genes and proteins expression [136], with overexpression of certain genes or activation of specific proteins directly contributing to resistance. For example, upregulation of HIST1H2BK is associated with enhanced anti‐apoptotic activity and membrane transport functions [137], whereas TRIM58 and TRIM37 influence anthracycline sensitivity through different mechanisms [138, 139].

Noncoding RNAs, particularly circRNAs and miRNAs, play crucial roles in ADM resistance. CircRNAs, such as circKDM4C and circUBE2D2 [140, 141, 142, 143], regulate the expression of resistance‐related genes by sponging miRNAs. Additionally, several miRNAs, including miR‐15b, miR‐23a, miR‐26a, and the miRNA‐449 family, modulate resistance by influencing‐related pathways and cell cycle factors [144, 145].

Inaddition, various biomolecules and cellular processes also contribute to ADM resistance in TNBC. Adipocyte‐derived factors and adipose‐derived mesenchymal stem cells (MSCs) induce ADM resistance through secreted factors and conditioned media [146, 147]. The obesity‐related protein FTO drives ADM resistance in breast cancer by activating the STAT3 pathway [148]. Epigenetic modifications, such as m6A methylation of HYOU1 regulated by METTL3/IGF2BP3 and m6A demethylation of FOXO1 mRNA mediated by ALKBH5, also play significant roles in resistance development [149, 150]. Galectin‐1 promotes TNBC resistance by activating the integrin β1/FAK/c‐Src/ERK/STAT3/survivin pathway [151].

Abnormal glucose metabolism is another crucial mechanism of ADM resistance in TNBC. Upregulation of key glycolytic enzymes, such as hexokinase, lactate dehydrogenase, and enolase [152], along with acylcarnitine/fatty acid oxidation and specific metabolites, supports the survival of TNBC cells under chemotherapy pressure [153]. Integrin β3 further promotes ADM resistance by inhibiting the pro‐apoptotic protein BAD [154]. High expression of FOXC1 is also associated with ADM resistance [155]. Heat shock protein β1 (HSPB1) enhances TNBC resistance to ADM by protecting cells from drug‐induced ferroptosis [156].

**FIGURE 4:**
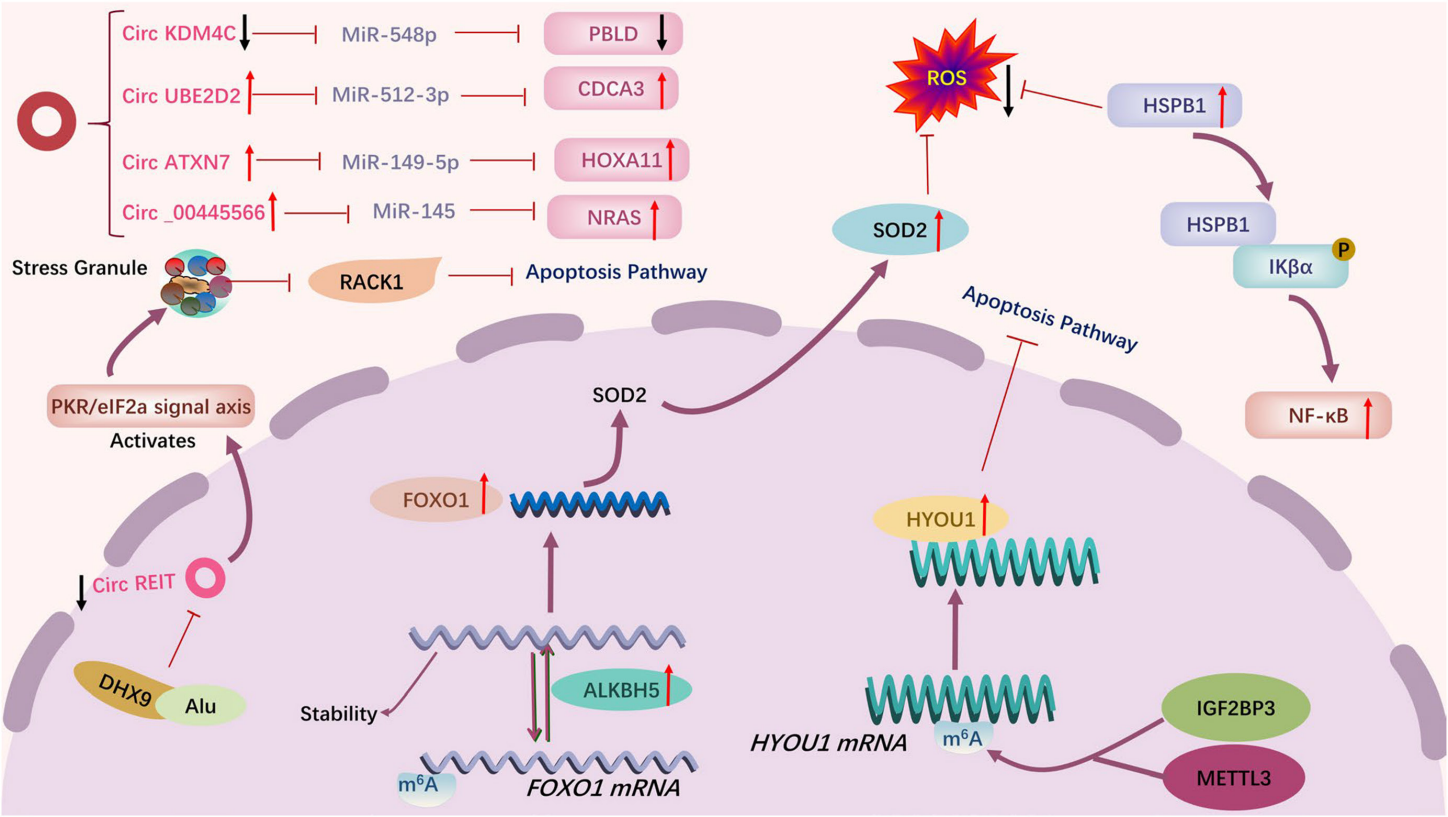
Mechanisms of chemoresistance to anthracycline‐based therapy in TNBC.

Furthermore, TMEPAI promotes EMT by altering the SMAD3 and PI3K/AKT pathways [157], whereas TNFSF13 triggers autophagy, leading to chemoresistance resistance [130]. Luo et al. identified a novel molecular mechanism wherein the lncRNA PVT1 enhances TNBC resistance to ADM by inhibiting the interaction between Keap1 and Nrf2, thus stabilizing Nrf2 protein [158] (Figure 4).

### 5.4 Major Causes of Chemotherapy Resistance in TNBC

Currently, multiple complex mechanisms underlying chemotherapy resistance in TNBC have been elucidated. These mechanisms primarily include [159, 160] (1) ATP‐binding cassette (ABC) transporters pump chemotherapeutic agents out of cells, reducing intracellular drug concentrations; (2) induction of cancer stem cells (CSCs) after neoadjuvant chemotherapy (NACT), which exhibit heightened resistance to chemotherapeutic agents; (3) hypoxic environments: not only leading to tumor microenvironment acidification and inadequate angiogenesis but also inducing CSC phenotypes, activating immune suppression pathways, and regulating apoptosis mechanisms to promote resistance; (4) tumor cells evading apoptosis; (5) abnormal expression of noncoding RNAs, which promote resistance by regulating various cellular processes; (6) epigenetic modifications, such as changes in DNA methylation and phosphorylated proteins; (7) abnormal activation of signaling pathways such as TGF‐β, Notch, Hedgehog, and Wnt/β‐catenin pathways playing key roles in CSC maintenance and excessive activation of NF‐κB, PTEN, and PI3K‐AKT–mTOR signaling pathways, all contributing to the resistance process in TNBC.

## 6 Recent Advances in the Treatment of TNBC

Chemotherapy, particularly neoadjuvant chemotherapy (NAC) based on anthracyclines and taxanes, is the standard treatment for locally advanced TNBC. However, only 30%–40% of patients achieve a complete pathological response (pCR), whereas the rest exhibit no response or disease progression (pNR), leading to poor prognoses [161]. Research has shown that combining chemotherapy with immune checkpoint inhibitors (ICIs) can enhance pCR rates [162]. For instance, in the IMpassion031 trial, the pCR rate for the combination of atezolizumab and chemotherapy was significantly higher than that of the placebo group [163]. Similarly, the IMpassion130 trial demonstrated that this combination improved both median progression‐free survival (PFS) and overall survival (OS) compared to the control group [164]. Although the IMpassion131 trial did not show a significant improvement in PFS for PD‐L1‐positive patients, the combination of pembrolizumab and chemotherapy exhibited better OS in patients with high PD‐L1 expression (CPS ≥ 10) in advanced TNBC [165]. Consequently, the effectiveness and prognosis of immunotherapy in advanced TNBC patients can potentially be predicted prior to treatment based on PD‐L1 expression.

Furthermore, ICIs can be combined with targeted therapies such as PARP inhibitors (PARPi) for cancers with HRD, proving especially effective for approximately 50% of TNBC patients with BRCA1/2 mutations. The FDA has approved four PARPi drugs, with olaparib and talazoparib being used for advanced or metastatic breast cancer with BRCA mutations [166, 167, 168]. In cases of BRCA1 deficiency or mutation, PARPi can also upregulate interferon responses via the STING pathway, increasing PD‐L1 expression and enhancing the immunogenicity of TNBC. Combining ICIs with anti‐angiogenic agents has also shown promise. For instance, low‐dose VEGFR2 antibodies stimulate CD8+ T cells and macrophages to secrete OPN, which promotes TGF‐β production and subsequently increases PD‐1 expression [169].

Given the malignancy, heterogeneity, and resistance of TNBC, monotherapy is often insufficient, making combination therapy the preferred approach [170]. In recent years, a growing body of research has assessed the efficacy of various combination immunotherapies, including combinations of chemotherapy, targeted therapy, and radiotherapy [171, 172]. Patients with PD‐L1+ status who receive standard chemotherapy combined with immunotherapy show favorable prognoses, whereas BRCA‐related mutation carriers also fare better following combination targeted therapies [173]. Future research will focus on immunosuppressive strategies in combination with chemotherapy, targeted therapies, and novel immunotherapies, including inhibitors targeting BRCA/PI3K/AKT/mTOR/CDK4/6 and adoptive immunotherapies (such as TIL and CAR‐T).

## 7 Conclusion

Triple‐negative breast cancer (TNBC) is a highly aggressive and malignant form of breast cancer characterized by high rates of recurrence and heterogeneity. The recognition of TNBC's diversity and heterogeneity is crucial for successfully analyzing the pathogenesis and therapeutic dependencies of this disease subtype. Advances in whole‐genome sequencing have enabled the identification of the most common mutated genes, the gene profiles most likely to drive cancer development, and the gene profiles associated with metastatic TNBC, which are valuable for pinpointing individual driver genes and mechanisms to determine therapeutic targets. Currently, standard chemotherapy remains the primary treatment for TNBC; however, these tumors often develop resistance to cytotoxic drugs. Therefore, elucidating the molecular drivers of chemotherapy resistance in TNBC is urgent and essential for improving prognosis and treatment. This review highlights the complexity of chemotherapy resistance in TNBC, which results from the interplay and synergistic induction of multiple factors and signaling pathways. Additionally, the review discusses biomarkers that can predict resistance to specific chemotherapeutic agents, which may guide treatment strategies for both early and late‐stage disease, ultimately improving patient survival. Lastly, it briefly summarizes the latest advancements in therapeutic strategies for TNBC.

## Author Contributions

**Yuhan Liu:** conceptualization (equal), investigation (lead), writing – original draft (lead). **Yuhan Zou:** investigation (supporting), resources (equal). **Yangli Ye:** conceptualization (equal), resources (equal). **Yong Chen:** supervision (lead), writing – review and editing (lead).

## Conflicts of Interest

The authors declare no conflicts of interest.

## Data Availability

Data sharing is not applicable to this article as no new data were created or analyzed in this study.
